# Metropolitan/nonmetropolitan differences of the impact of COVID‐19 on cancer survivors' care

**DOI:** 10.1111/jrh.70061

**Published:** 2025-07-30

**Authors:** Whitney E. Zahnd, Jason T. Semprini, Robin C. Vanderpool, Sarah H. Nash, Erin L. Van Blarigan, Mindy C. DeRouen, Angela L. W. Meisner, Chuck Wiggins

**Affiliations:** ^1^ Department of Health Management and Policy College of Public Health University of Iowa Iowa City Iowa USA; ^2^ Department of Public Health College of Health Sciences Des Moines University West Des Moines Iowa USA; ^3^ Health Communication and Informatics Research Branch Behavioral Research Program Division of Cancer Control and Population Sciences National Cancer Institute Rockville Maryland USA; ^4^ Department of Epidemiology and Iowa Cancer Registry College of Public Health University of Iowa Iowa City Iowa USA; ^5^ Department of Epidemiology and Biostatistics University of California San Francisco San Franscico California USA; ^6^ Greater Bay Area Cancer Registry San Francisco California USA; ^7^ University of New Mexico Comprehensive Cancer Center and New Mexico Tumor Registry Albuquerque New Mexico USA

**Keywords:** cancer, cancer survivorship, COVID‐19 pandemic, preventive care, rural health, telehealth

## Abstract

**Purpose:**

To evaluate pandemic‐related changes in cancer‐related care for cancer survivors residing in nonmetropolitan and metropolitan areas.

**Methods:**

We used data from the Health Information National Trends‐Surveillance Epidemiology End Results (HINTS‐SEER) survey administered to cancer survivors from the Greater San Francisco Bay Area, Iowa, and New Mexico between January and August 2021. Respondents were queried on changes to their cancer‐related care, including treatment, follow‐up appointments, and routine cancer screening/preventive care. We calculated weighted percentages and Rao‐Scott chi‐square tests for reported differences between nonmetropolitan and metropolitan areas.

**Findings:**

Compared to survivors residing in metropolitan areas, a higher proportion of those in nonmetropolitan areas reported that their cancer treatment or follow‐up appointments were unaffected by the pandemic (38.6% vs 28.1%; *P* = .008). Survivors in metropolitan areas experienced more of a shift in cancer treatment or follow‐up appointments to telehealth (12.5% vs 5.7%, *P* = .003), but there was no difference in appointment cancellations. More survivors residing in metropolitan versus nonmetropolitan areas reported shifts to telehealth for preventive care (8.2% vs 2.9%, *P* = .005). There was no difference across nonmetropolitan and metropolitan survivors reporting that cancer‐related care was cancelled, that routine cancer screening or preventive care was unaffected by the pandemic, or that providers discussed COVID‐19 risks.

**Conclusions:**

Survivors in nonmetropolitan compared to metropolitan areas had less perceived change in cancer follow‐up and treatment schedules. It will be important to assess whether shifts in follow‐up and preventive care to telehealth for cancer survivors in need of care during the COVID‐19 pandemic affect their long‐term outcomes.

## INTRODUCTION

With advances in early detection and treatment, the number of cancer survivors has grown in the last several decades; there were more than 18 million cancer survivors in the United States in 2022.^1^ After initial treatment, cancer survivors continue to seek care to address long‐term side effects, psychosocial issues, screening for additional cancers, and surveillance for recurrent cancers. Rural residents have higher cancer incidence rates and poorer cancer prognosis compared to their urban counterparts.^2^, ^3^ Further, compared to their urban counterparts, cancer survivors residing in rural areas face additional health‐related challenges, including worse self‐reported health, more noncancer comorbidities, and greater likelihood of uninsured status.^4^ These factors may necessitate greater need for health care services compared to their urban counterparts and reflect increased barriers to receiving these services. Survivors residing in rural areas report less satisfaction with the ease in receipt of health care services.^5^ Additionally, those living in rural areas have less access to hospitals that provide oncology and telehealth services, which may make it more difficult to access important survivorship services.^6^

As the COVID‐19 pandemic progressed, its mortality impact fell disproportionately on rural (nonmetropolitan) populations, particularly during the winter peak of late 2020 and early 2021.^7^ The pandemic also had an impact on the provision of cancer care across the continuum, from screening to treatment to survivorship care. Studies show that Medicare‐certified rural health clinics reduced cancer screening and prevention services and experienced other notable disruptions (eg, temporary closures, provider burnout, and turnover) during the first year of the pandemic while expanding telehealth services.^8^ Cancer survivors were more vulnerable to complications due to SARS‐COV2 infection. Therefore, receipt of survivorship services and follow‐up care while reducing COVID‐19 exposure—such as through use of telehealth—was critical. This was particularly key when COVID‐19 vaccines were introduced in 2021. However, disparities in accessibility of health care services generally for people residing in nonmetropolitan and metropolitan areas have been noted; survivors residing in rural communities were less likely than those in urban areas to have access to and to use telehealth services for all health care services in 2020 and 2021.^9^ Yet, pandemic‐related changes to cancer care for survivors residing in rural compared to urban areas are unknown. Therefore, our objective was to examine rural and urban differences in the impact of the COVID‐19 pandemic on cancer‐related health care visits among cancer survivors. Understanding how macro‐level stressors (eg, disease outbreaks, natural disasters, economic recessions) affect cancer‐related care for cancer survivors will inform intervention points to reduce disparities for vulnerable groups, including residents of rural areas.

## METHODS

We accessed the Health Information National Trends Survey‐Surveillance Epidemiology and End Results (HINTS‐SEER) dataset. HINTS is a nationally representative survey that has been administered regularly by the National Cancer Institute since 2003. HINTS questions focus on cancer and health information, communication preferences, attitudes, and knowledge, as well as health‐related behaviors. To date, cancer survivors have typically comprised 9% of HINTS’ study samples. Therefore, NCI conducted a pilot project in 2021, leveraging SEER‐Program cancer registries. NCI assessed cancer registries' interest in assisting in recruiting cancer survivors for this survey with a goal of ensuring racial/ethnic, rural, and regional diversity.^10^ Ultimately, the Greater San Francisco Bay Area in California, Iowa, and New Mexico agreed to participate and sampled cancer survivors over the age of 18 years and who had a cancer diagnosis prior to 2018 (to ensure complete and accurate collection of information on cancer treatment and follow‐up data).^11^ Overall response was 6.3% for Iowa, 24.1% for the Greater Bay Area, and 24.6% for New Mexico.^10^ Variations in response rates were driven due to differences in consent processes (ie, active vs passive consent). As an implicit stratification sampling approach was used in general, HINTS‐SEER respondents were similar in demographic characteristics to the survivor population from which they were sampled. Surveys were administered via mail between January and August of 2021.

Survey participants were queried on the impact of the pandemic on 2 types of care: (1) appointments of cancer treatment and cancer‐related follow‐up; and (2) appointments of routine cancer screening or preventive care (n = 1,178 respondents had valid responses to these questions). They then selected all that applied from the following options: no appointments scheduled, appointments unaffected, some or all appointments cancelled or delayed, or some or all appointments shifted to phone or telehealth. Additionally, participants were asked if their health care provider had discussed or provided information about the risk of COVID‐19 complications due to their cancer history, with response options as yes, no, or do not know.

We also considered sociodemographic (ie, sex, age, race/ethnicity, insurance status, household income, educational attainment) and cancer characteristics (ie, cancer type, age at diagnosis, time since diagnosis). Participants were classified as residing in a metropolitan or nonmetropolitan county based on Urban Influence Codes (UIC; 1‐2 for metropolitan and 3‐12 for nonmetropolitan), which categorize counties by their population size and urbanicity.^12^

To examine nonmetropolitan/metropolitan differences in sociodemographic characteristics and changes in cancer care during the COVID‐19 pandemic, we calculated weighted percentages and Rao‐Scott chi‐square tests. All analyses were performed in SAS 9.4 and accounted for the sampling and complex survey design by using jackknife replication variance estimation procedures congruent with NCI analytical guidance, and to ensure appropriate inferences from the sample, correcting for response and nonresponse biases.^10^

## RESULTS

The weighted percentage of nonmetropolitan survivors was 17.2% (Table 1). Nonmetropolitan and metropolitan respondents were similar on sociodemographic characteristics, except for educational attainment.

**TABLE 1:** Sociodemographic characteristics of 1,178 nonmetropolitan and metropolitan cancer survivors, Surveillance Epidemiology and End Results‐Health Information National Trends Survey study (2021).

	Nonmetropolitan weighted % (n = 269)	Metropolitan weighted % (n = 909)	*P*‐value
Age			
<50	4.5%	5.7%	.30
50‐64	27.9%	21.8%	
65‐74	27.6%	31.9%	
75+	40.0%	40.5%	
Race/ethnicity			
Asian	^**^	9.2%	——–
Hispanic	11.5%	12.1%	
Non‐Hispanic Black	^**^	3.4%	
Non‐Hispanic Other	^**^	1.3%	
Non‐Hispanic White	87.9%	73.9%	
Sex			
Male	45.4%	45.0%	.99
Female	54.6%	55.0%	
Marital status			
Married/living as married	75.7%	68.2%	.14
Divorced/separated	8.6%	12.6%	
Widowed	11.2%	12.3%	
Single, never married	7.5%	7.2%	
Education			
Less than high school	2.3%	3.3%	<.001
High school	14.3%	9.9%	
Technical college/some college	37.2%	23.9%	
College graduate	41.4%	62.7%	
Time since diagnosis			
≤ 5 years	20.2%	17.7%	.78
6‐10 years	22.2%	24.3%	
11+ years	57.5%	58.0%	

*Note*: Some weighted percentages may be based on smaller sample sizes due to missing responses to some questions.

** Data are suppressed due to being based on sample sizes <25 respondents. *P*‐values generated from Rao‐Scott chi‐square tests.

Compared to survivors residing in metropolitan areas, those in nonmetropolitan areas were more likely to report that their cancer treatment or follow‐up appointments were unaffected by the pandemic (28.1% for metropolitan vs 38.6% for nonmetropolitan; *P* = .008; Table 2). More survivors residing in metropolitan areas experienced a shift in treatment or follow‐up appointments to telehealth compared to nonmetropolitan areas (12.5% vs 5.7%, *P* = .003), but there was no difference in appointment cancellations. More survivors residing in metropolitan than nonmetropolitan areas also reported shifts to telehealth for routine cancer screening/preventive care (8.2% vs 2.9%, *P* = .005). There was no metropolitan/nonmetropolitan difference in the percentage of cancer survivors who reported their routine cancer screening/preventive care was unaffected by the pandemic (38.1% vs 44.5%, *P* = .09) or experienced cancellations (16.2% vs 13.4%, *P* = .20). The percentage of survivors whose providers discussed the risk of COVID‐19 complications due to cancer history was low for survivors residing in both metropolitan (14.5%) and nonmetropolitan (12.1%) areas (*P* = .34).

**TABLE 2:** Impact of COVID‐19 pandemic on cancer care among nonmetropolitan and metropolitan cancer survivors; Surveillance Epidemiology and End Results‐Health Information National Trends Survey study (2021).

	Nonmetropolitan weighted % (n = 269)	Metropolitan weighted % (n = 909)	*P*‐value
***Appointments related to cancer treatment or follow‐up***			
COVID‐19 pandemic did not affect appointments, yes	38.6%	28.1%	.008
Appointment cancelled due to COVID‐19 pandemic, yes	6.0%	5.7%	.80
Some/all appointments were shifted to phone or telehealth, yes	5.7%	12.5%	.003
***Appointments related to routine cancer screening or preventive care***			
COVID‐19 pandemic did not affect appointments, yes	44.5%	38.1%	.09
Appointment cancelled due to COVID‐19 pandemic, yes	13.4%	16.2%	.20
Some/all appointments were shifted to phone or telehealth, yes	2.9%	8.2%	.005
***Provider discussion about COVID‐19 risk***			
Health care provider discussed COVID‐19 risk or complications due to cancer history, yes	12.1%	14.5%	.34

*Note*: *P*‐values generated from Rao‐Scott chi‐square tests.

## DISCUSSION

The current study examined the impact of the COVID‐19 pandemic on cancer follow‐up and treatment among survivors residing in nonmetropolitan compared to metropolitan areas using a representative sample of cancer survivors in 3 states. Survivors residing in nonmetropolitan areas reported fewer pandemic‐related changes to cancer‐related follow‐up and treatment, as well as routine screening and preventive care. Provider discussion of COVID‐19 risk and potential complications due to cancer history was low among both groups.

A higher proportion of survivors residing in metropolitan areas had cancer treatment, follow‐up, or preventive appointments affected by the pandemic; in particular, this was reflected in shifts in appointments to telephone or telehealth. This finding corroborates other studies showing higher overall telehealth usage among survivors residing in metropolitan compared to nonmetropolitan areas, though our study examines the COVID‐19 impact specifically on cancer‐related care.^9^, ^13^ Contributing factors to this difference in telehealth use are unclear, but may be driven by myriad factors. Prior to the pandemic, studies showed that rural hospitals were less likely to have telehealth and oncology services, which may have persisted during the pandemic, making it more challenging for rural patients to access care either in‐person or via telehealth.^6^ Consequently, patients residing in rural areas may not have had the opportunity to receive follow‐up care via telehealth. Studies show that telehealth implementation was a barrier for rural health clinics, affecting their ability to make prevention and screening services available during the pandemic.^14^ Although there was no difference in treatment and follow‐up appointment cancellation among survivors residing in nonmetropolitan compared to metropolitan areas, it is important that those residing in nonmetropolitan areas have access to care both in‐person and telehealth. This broad access enables them to receive important services while reducing exposures to infectious diseases they may be exposed to in a health care setting. However, a 2022 survey of Iowans, one of the HINTS‐SEER states, found that people residing in rural areas were less concerned about COVID‐19 risk and exhibited fewer behavioral changes, such as social distancing and masking, and fewer disruptions in daily living compared to urban individuals.^15^, ^16^ Thus, rural residents may be more likely than urban residents to choose in‐person care, even if telehealth options are available. Additionally, while findings did not show metropolitan and nonmetropolitan differences in appointment cancellations related to the pandemic for preventive and screening visits, continued surveillance is important to ensure patients are receiving necessary follow‐up and preventive care to ensure optimal survivorship and early detection of new, primary cancers.

Nonmetropolitan and metropolitan cancer survivors did not differ in the frequency of reported discussions with their providers about elevated COVID‐19 risk due to their cancer history, as few respondents in both groups reported receiving information about COVID‐19 risk. This represents an opportunity for better patient education for cancer patients and survivors, regardless of geography. Prior to the widespread availability of COVID‐19 vaccines, studies showed that patients with a history of cancer who also had COVID‐19 had worse outcomes (ie, hospitalization, death) compared to others with either solely COVID‐19 or cancer, which also varied by cancer type and patient race/ethnicity.^17^ This was particularly true for those with a recent cancer diagnosis (ie, within the past year). Since COVID‐19 vaccines became broadly available, disparities in vaccination uptake have widened between rural and urban communities.^18^ In a post‐pandemic world, it remains important for rural cancer patients and survivors, who are at greater risk for complications and have less access to and uptake of vaccination protecting against hospitalization and death, to be informed about the ongoing risk of COVID‐19 and related necessary precautions as well as emerging infectious diseases that may disproportionately affect cancer survivors.

Our study found more shifts to telehealth among metropolitan survivors during the pandemic, which may have been driven by limited access to telehealth and broadband or by patient choice.^19^ The pandemic exists as an example of the potential impact of macro‐level stressors on health care quality and access for populations that already experience inequities, including cancer survivors residing in rural areas.^20^ Thus, it is important to assess the capacity of health care systems to respond to patient needs during macro‐level crises, including natural disasters and socioeconomic recessions.^21^ To avoid increasing health inequities for vulnerable populations in the future, we must identify strategies to foster resilience in health care systems.^21^

HINTS‐SEER is an important dataset that surveys cancer survivors leveraging cancer registries. However, there are important limitations. First, respondents surveyed from cancer registries are at least 2 years post‐diagnosis, and thus, many were not currently receiving cancer treatment during the pandemic. Additionally, the HINTS‐SEER dataset only includes 3 cancer registries, which may affect the generalizability of findings beyond cancer survivors in these catchment areas, as well as a resultant small sample size. However, the 3 registries represent a mix of urban and rural communities across different regions of the country, and the population sampled from each registry was representative of that registry's population of cancer survivors. Also, the survey did not include questions about whether cancer survivors were offered telehealth as an option for care, and if so, whether they chose not to use it, making the drivers of appointment modality unclear.

## CONCLUSIONS

We examined the impact of the COVID‐19 pandemic on nonmetropolitan and metropolitan cancer survivors’ receipt of follow‐up care, treatment, and routine care. Although there were no differences in appointment cancellation, we found that metropolitan survivors were more likely to have an appointment switch to a telehealth or telephone appointment. We also found that provider discussions of COVID‐19 risks were low regardless of geography. With a larger risk for complications due to COVID‐19 among cancer survivors and with lower uptake of vaccination among nonmetropolitan populations, education of rural populations and cancer survivors is important to reduce the risk of poor survivorship outcomes.

## CONFLICT OF INTEREST STATEMENT

The opinions expressed by the authors are their own, and this material should not be interpreted as representing the official viewpoint of the US Department of Health and Human Services, the National Institutes of Health, or the National Cancer Institute. Zahnd currently serves as the chair of the editorial board of the *Journal of Rural Health*. This study received a nonhuman subjects’ determination from the University of Iowa Institutional Review Board.
